# The A.B.C. of Vitamins.—II

**Published:** 1922-04

**Authors:** Harold Scurfield

**Affiliations:** (formerly Medical Officer of Health, Sheffield)


### THE A.B.C. OF VITAMINS.—II.

**By HAROLD SCURFIELD, M.D., D.P.H. (formerly Medical Officer of Health, Sheffield).**

" A pint of milk, egg, orange and greens
Will give you your daily vitamins "

was the burden of the last article, with the notes that tomatoes are cheap when oranges are dear, that uncooked greens such as lettuce and watercress are the best, that dried eggs are as good as new laid, and that dried milk, especially if it is the product of cows fed on green food, is a satisfactory form in which to take the milk.

Although the foods mentioned are not those which most people tire of readily, there are people who cannot eat eggs, and people who think milk does not agree with them, and, in any case, it is well for the sake of variety to have reserves for our supplies of Vitamins A B and C. If, for any reason, we fail to get our quantum of milk, eggs and greens, it ought to be possible to make up any deficiency in vitamin A from butter, beef-fat, mutton-fat and herrings. It is well, too, to bear in mind that the offal usually contains more of vitamins A and B than the meat itself, and that liver, kidneys, heart, brain and sweetbreads make a useful change from a joint.

As a source of vitamin A, cod-liver oil is our most valuable stand-by. The fame of cod-liver oil has undergone vicissitudes. It may have come to us from the Vikings. Belief in it waned, at times during the nineteenth century, and it became fashionable to regard extra butter as being just as good. The vitamin research has restored cod-liver oil to its high place, and one might adapt the old whist rule of " when in doubt, play trumps " to the giving of cod-liver oil in the case of anyone suspected of having been starved of vitamin A. A word of caution is necessary. One does not always want to give malt with cod-liver oil. Malt does not pre-digest the oil, and to give six or seven teaspoonsful of a mixture of malt and oil in order that the patient may get one teaspoonful of oil is a very clumsy proceeding. Cod-liver oil is an easily digested fat, and some people take it quite readily by itself. Those who dislike it can easily obtain a palatable 50 per cent. emulsion, so that two teaspoonsful of the emulsion contain one teaspoonful of the oil.

As regards reserves for vitamin B, if we fail to get our allowance of milk and egg, extract of yeast is a very convenient source of supply. When the war had been in progress some years, we were allowing tons of yeast to be discharged into the sewers, while the Germans were actually growing yeast on account of its food value. A correspondence with Government departments on this subject introduced me to Marmite. I have no doubt there are other extracts of yeast on the market now. Since my introduction to Marmite we have never been without it, and have found it most useful for the making of soups, gravies, stews, &c. Anyone using it for the first time will be surprised at its resemblance to a meat extract. Marmite was in use before its high content of vitamin B was known. Now that the discovery has been made, one hopes that extract of yeast will come into common use and become much cheaper. Even although we are not at war, yeast is too valuable an article to be thrown into the sewers. White flour contains no vitamin B, but whole-meal does. Dr. Hamill suggests that the yeast used in baking may replace the B lost in the refining of the flour. The stories told by the Medical Research Committee suggest that the yeast used in baking is not a sufficient safeguard in the case of persons living on a restricted diet. Thus the Newfoundlanders developed beri-beri owing to the substitution of white bread for brown ; the Norwegian sailors suffered similarly as the result of the substitution of white bread for wholemeal rye biscuits, and the British troops in Mesopotamia and Kut fed on white bread were also attacked by beri-beri. Presumably all these breads were made with yeast. Dietaries in this country may be seldom so restricted as to cause the danger of beri-beri, but they may be sufficiently short of B to retard growth in the young, and cause obscure symptoms at any age. If we want to be at the top of our form, we shall do well to eat wholemeal bread, to use extract of yeast in cooking and bear in mind the offal already mentioned, which is rich in B.

As regards reserves for vitamin C, which prevents scurvy, green vegetables give us a wide range and almost all fresh fruit has some anti-scorbutic value.

An interesting discussion has been going on in the " Times " lately on the feeding of schoolboys. Letters from old generals, stating how exceedingly they have flourished subsequently to a very primitive dietary in their schooldays, prove nothing. We have to face the revelations of the recruiting statistics, and the facts that there is wide-spread dental decay, and that the national physique is not what it might be. It is possible that the school dietaries will not all come out well by the Mellanby-Hamill standard. There will probably be a shortage of eggs and milk. The house-master's garden as a source of lettuce, cabbage, &c., may become very important. There seems to be no reason why oranges should not be included in a school dietary if they are bought in a wholesale fashion. One would like to see " tuck shops " restricted to the sale of fruit.

Perhaps the change of our dietary involved in the modern craze for sweets and sugar is not always realised. In 1700 1 cwt. of cane sugar per annum was consumed by 30 persons in these islands ; in 1800 1 cwt. was enough for 5 persons ; and in 1900 1 cwt. was only enough for one person. If sugar had only been substituted for starch it would not so much matter, but we know that in the form of vitamin-less jam it has been substituted for butter.

The vitamin research work ought to give a stimulus to the cultivation of allotments and the growing of lettuces, watercress and tomatoes, to the keeping of poultry, to the importation of oranges and their increased production in our Colonies, and to the greatly increased use of fruit of all kinds. One hopes, too, that the call for economy will not induce the Government to take any steps which may curtail our ridiculously low consumption of milk per head.

